# Genome-Wide Occupancy of SREBP1 and Its Partners NFY and SP1 Reveals Novel Functional Roles and Combinatorial Regulation of Distinct Classes of Genes

**DOI:** 10.1371/journal.pgen.1000133

**Published:** 2008-07-25

**Authors:** Brian D. Reed, Alexandra E. Charos, Anna M. Szekely, Sherman M. Weissman, Michael Snyder

**Affiliations:** 1 Department of Molecular, Cellular, and Developmental Biology, Yale University, New Haven, Connecticut, United States of America; 2 Department of Genetics, Yale University School of Medicine, New Haven, Connecticut, United States of America; 3 Molecular Biophysics and Biochemistry Department, Yale University, New Haven, Connecticut, United States of America; The Jackson Laboratory, United States of America

## Abstract

The sterol regulatory element-binding protein (SREBP) family member SREBP1 is a critical transcriptional regulator of cholesterol and fatty acid metabolism and has been implicated in insulin resistance, diabetes, and other diet-related diseases. We globally identified the promoters occupied by SREBP1 and its binding partners NFY and SP1 in a human hepatocyte cell line using chromatin immunoprecipitation combined with genome tiling arrays (ChIP-chip). We find that SREBP1 occupies the promoters of 1,141 target genes involved in diverse biological pathways, including novel targets with roles in lipid metabolism and insulin signaling. We also identify a conserved SREBP1 DNA-binding motif in SREBP1 target promoters, and we demonstrate that many SREBP1 target genes are transcriptionally activated by treatment with insulin and glucose using gene expression microarrays. Finally, we show that SREBP1 cooperates extensively with NFY and SP1 throughout the genome and that unique combinations of these factors target distinct functional pathways. Our results provide insight into the regulatory circuitry in which SREBP1 and its network partners coordinate a complex transcriptional response in the liver with cues from the diet.

## Author Summary

Transcription factors (TFs) are DNA-binding proteins that regulate the transcription of their target genes. TFs typically bind in proximity to the transcription start sites of their target genes in a region called the promoter. SREBP1 is a TF that increases the transcription of numerous genes involved in cholesterol and fat metabolism and has been linked to diet-related diseases such as insulin resistance and type 2 diabetes. Using microarray technology, we identified all of the promoters in the human genome that are bound by SREBP1 and two associated TFs called NFY and SP1 in a human liver cell line. Our findings greatly expand the number of genes and biological pathways that may be regulated by SREBP1 and reveal that different combinations of SREBP1 and its partners preferentially target genes involved in different pathways. Thus, in contrast to traditional studies that focus on individual genes, we have used a genomics approach to provide a novel global view of the regulatory circuitry in which SREBP1 and its partners coordinate a transcriptional response in the liver with cues from the diet.

## Introduction

The transcription factor SREBP1 is a key regulator of the transcription of numerous genes that function in the metabolism of cholesterol and fatty acids [1]. Alternative promoter usage gives rise to two nearly identical isoforms of SREBP1—SREBP1a and SREBP1c—that differ from each other at a few N-terminal amino acids encoded by different first exons [2]. Both isoforms begin as ER membrane-bound precursors and require proteolytic cleavage to release N-terminal fragments that function in the nucleus [3]. Levels of nuclear SREBP1a increase in response to cholesterol depletion through a sterol-sensing pathway that determines the rate of cleavage [3]. In contrast, levels of nuclear SREBP1c increase in response to insulin signaling [4],[5] and stimulation of the nuclear receptor LXRα [6]—inputs that upregulate the transcription of *SREBP1c*. SREBPs are weak transcriptional activators on their own and interact with their target promoters in cooperation with additional regulators, most commonly including one or both of the transcription factors NFY and SP1 [7]–[9].

Excessive accumulation of fatty acids in the liver is associated with a number of diet-related diseases such as insulin resistance, metabolic syndrome, and type 2 diabetes. The de novo synthesis of fatty acids (lipogenesis) in the liver is induced by the insulin signaling pathway. Numerous studies based on the analysis of single genes have demonstrated that SREBP1 upregulates the transcription of lipogenic genes, primarily through the insulin-responsive SREBP1c isoform which predominates in the liver [1]. Although these studies have identified a number of important lipogenic genes that are regulated by SREBP1, our understanding of the targets of SREBP1 involved in lipid metabolism is incomplete and the role of SREBP1 in other pathways that may contribute to diet-related disease states is poorly understood. Furthermore, our understanding of the manner in which SREBP1 selects and regulates its target genes in combination with other transcriptional regulators is limited.

**Figure 1:**
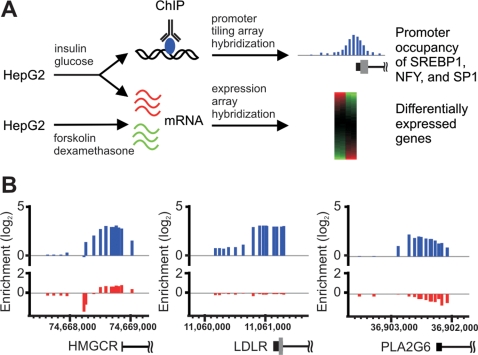
Study design and ChIP enrichment at representative SREBP1-occupied promoters. A) Overall scheme to identify targets of SREBP1, NFY, and SP1 in HepG2 cells and to investigate the regulation of SREBP1 targets under opposing nutrient conditions. B) SREBP1 targets were identified using ChIP-chip with promoter tiling arrays; three representative SREBP1-occupied promoters are shown. Smoothed log_2_ ChIP enrichment values of tiled DNA probes are represented by vertical bars. Upper plots represent ChIP enrichment with an SREBP1-specific antibody whereas lower plots represent ChIP enrichment with rabbit IgG used as a negative control. Array hybridizations of unenriched (input) DNA were used as a reference for calculating ChIP enrichment. Chromosomal positions are indicated on the x-axis. Gene structure is shown to scale below each plot with 5′-UTRs (black boxes), exons (grey boxes) and introns (black lines) indicated.

In the present study, we sought to further understand the function of SREBP1 and how it operates with other regulators by globally mapping its target genes and those of its associated factors NFY and SP1 in human hepatocarcinoma (HepG2) cells using ChIP-chip. In addition, we sought to correlate SREBP1 occupation with changes in gene expression induced under conditions in which SREBP1 is activated and repressed. The overall scheme is depicted in Figure 1A.

## Results/Discussion

### Genome-Wide Identification and Characterization of SREBP1 Binding Sites in Human Promoters

**Figure 2:**
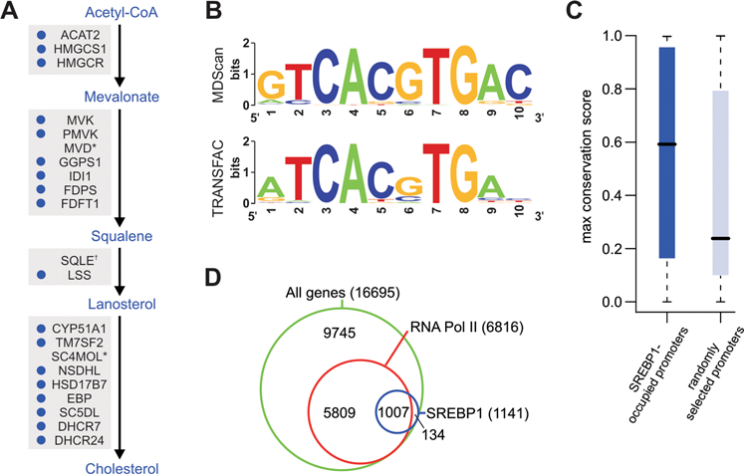
Key features of SREBP1 target genes and binding sites. A) The cholesterol biosynthesis pathway with key intermediates indicated in blue text and enzymes represented by their gene symbols. Blue circles indicate genes identified as SREBP1 targets by ChIP-chip. *The promoters of mevalonate pyrophosphate decarboxylase (MVD) and sterol-C4 methyl oxidase (SC4MOL) were not represented on the promoter array. ^†^The squalene epoxidase (SQLE) promoter narrowly missed our significance thresholds; we confirmed SREBP1 binding by ChIP-qPCR. B) The top-scoring motif discovered in an unbiased analysis of SREBP1-occupied promoter sequences using MDScan [12] (top) compared to the known E-box-containing SREBP1 consensus from the TRANSFAC [14] database (bottom). C) Evolutionary conservation of DNA sequences matching the discovered E-box motif. Boxplots represent the median and interquartile range of the maximum phastCons conservation scores [16] of 10 bp matching sequences located in the set of SREBP1-occupied promoters (dark blue) or in an equally-sized set of random promoters (light blue). The conservation score (ranging from 0 to 1) represents the probability that a base is in a conserved element [16]. The increase in conservation of matching sequences in SREBP1-occupied promoters is significant (*P* = 3.0×10^−3^ using a Wilcoxon rank-sum test). D) Venn diagram illustrating the overlap among SREBP1-occupied genes, RNA Pol II-occupied genes, and the total set of genes represented on the promoter tiling array.

The physiological levels of hepatic SREBP1c are downregulated in the fasted state and dramatically upregulated upon refeeding with high carbohydrates [10]. Therefore, to identify the complete repertoire of hepatic SREBP1 target genes using ChIP-chip, we treated HepG2 cells with insulin and glucose—conditions that mimic the high-carbohydrate refeeding response. We then performed chromatin immunoprecipitation with a well-characterized antibody that recognizes both SREBP1 isoforms due to their nearly identical primary structure. Since both isoforms are present in the nucleus under these conditions, the binding profiles generated include targets of both SREBP1a and SREBP1c. After amplification and fluorescent labeling, the immunoprecipitated DNA was hybridized to NimbleGen human promoter tiling arrays. These arrays contain 50-mer oligonucleotide probes tiled at 100 bp resolution from 1200 bp upstream to 300 bp downstream of the transcriptional start sites of approximately 16,700 annotated human genes. Examples of the enrichment profiles generated at representative SREBP1 target promoters are shown in Figure 1B. Statistical analysis of four replicate experiments using a hidden Markov model approach [11] provided evidence for SREBP1 occupation at the promoters of 1141 genes (Tables S1 and S2). The interaction dataset exhibited a striking enrichment (approximately 9-fold) for known targets of SREBP1 (*P* = 1.4×10^−29^, hypergeometric test)—37 genes occupied by SREBP1 in our analysis have been previously characterized as SREBP1 targets in the liver (Table S3). This subset includes 18 out of 19 represented SREBP1 target genes in the cholesterol biosynthesis pathway (Figure 2A) as well as known target genes involved in fatty acid biosynthesis and the pentose phosphate pathway (a pathway that supplies the NADPH required for lipid synthesis).

To determine the accuracy of our ChIP-chip results, we selected a random set of 19 putative SREBP1 binding sites and performed quantitative PCR using site-specific primers (Table S4). Binding was confirmed for 17 of these sites (Figure S1).

We next performed an unbiased search for DNA sequence motifs representing protein-DNA interaction sites in the set of SREBP1 target sequences using the program MDScan [12]. The top-scoring motif discovered in this search (Figure 2B) was 10 bp in length and contained an E-box consensus CANNTG known to bind basic helix-loop-helix transcription factors such as SREBP1 [13]. Indeed, comparison of the discovered motif to the TRANSFAC [14] database using a motif comparison and alignment algorithm [15] identified the known SREBP1 consensus E-box motif as the most similar match (Figure 2B and Figure S2). To investigate the evolutionary sequence conservation of SREBP1 binding sites, we scanned the set of SREBP1-occupied promoters and a set of randomly selected promoters for matches to the discovered motif. The matching 10 bp DNA sequences identified in SREBP1 target promoters exhibited significantly higher levels of conservation across vertebrate species (*P* = 3.0×10^−3^) based on their phastCons conservation scores [16] than matching sequences in the randomly selected promoter set (Figure 2C). Thus, the binding sites and motifs identified are likely to have a functional role in vivo.

To determine the fraction of SREBP1-occupied genes that is potentially transcribed in HepG2 cells, we performed ChIP-chip with an antibody that recognizes the initiating form of RNA polymerase II. Our results revealed that 43% (*n* = 7070) of the genes on the promoter array were occupied by RNA polymerase II (Figures S1 and S2). As expected, the vast majority of SREBP1 target genes (88%, *n* = 1007) were also occupied by RNA polymerase II (Figure 2D).

### Novel and Expanded Functional Roles for SREBP1

To explore the cellular processes that are regulated by SREBP1, we examined the functional categories associated with SREBP1 targets genes based on annotations in the Gene Ontology (GO) [17] database and manual curation and identified enriched GO categories using the BiNGO program [18] (Figure S3 and Table S5). Genes associated with lipid metabolism were highly enriched in the interaction dataset (*P* = 1.5×10^−4^), consistent with the known functions of SREBP1. Inspection of these genes revealed 41 novel SREBP1 targets (Table S6), considerably expanding the repertoire of SREBP1-regulated genes involved in lipid metabolism. One important target that we identified in this category, *ADIPOR2*, encodes the primary liver receptor for the insulin-sensitizing adipocytokine adiponectin [19]. Intriguingly, *ADIPOR2* expression is inversely regulated by insulin in hepatocytes [20], suggesting that SREBP1 may play a repressive role at the *ADIPOR2* promoter. Other important targets include endothelial lipase (*LIPG*), a key player in HDL metabolism [21], ethanolamine kinase 1 (*ETNK1*), an enzyme that catalyzes the first committed step in phosphatidylethanolamine synthesis, and sterol O-acyltransferase 2 (*SOAT2*), an enzyme responsible for cholesterol esterification in the liver [22].

Further investigation of the functions of novel SREBP1 target genes suggested that SREBP1 plays a more expansive role in a number of other processes in which it has been previously implicated. For example, SREBP1 is known to regulate the expression of *IRS2* [23] and *PIK3R3* [24], components of the insulin signaling pathway. We identified promoter interactions with several additional components of this pathway, including *AKT1S1*, *AKT2*, *GRB2*, *GSK3A*, *MEKK2*, *PRKCQ* (also known as *PKCθ*), and *S6K1*. These interactions suggest that SREBP1 is involved in complex feedback regulation of insulin signaling and implicate SREBP1 in additional branches of the insulin signaling pathway such as the regulation of protein synthesis. Additionally, recent studies have identified SREBP1a as a transcriptional activator of the cyclin-dependent kinase inhibitor gene *CDKN1A* (also known as *p21*)—an interaction that likely provides a crucial link between the synthesis of membrane lipids and cell proliferation [25]. Our findings support the role of SREBP1 in linking these processes, as 60 genes associated with cell cycle regulation based on GO annotation were identified among SREBP1 targets. Important cell cycle regulators occupied by SREBP1 include *ABL1*, *CCNG2*, *CDC25C*, *CDK2*, and *E2F4*. Finally, the targets of SREBP1 were enriched (*P* = 1.1×10^−3^) for genes involved in cellular respiration (e.g. *CYCS*, *IDH3G*, *SUCLA2*, *SDHB*, and *MDH1*), providing insight into the role of SREBP1 in energy metabolism and expanding on the known regulatory interaction of SREBP1 with the mitochondrial aconitase gene *ACO2* [26].

The list of SREBP1 target genes was also enriched for genes in a variety of GO categories that have not previously been associated with SREBP1, including RNA processing (*P* = 1.1×10^−11^), protein transport (*P* = 8.1×10^−6^), and DNA metabolism (*P* = 3.0×10^−2^). Taken together, our results suggest that SREBP1 may be involved in coordinating the activity of a wide range of cellular pathways with nutrient availability at the transcriptional level.

### Gene Expression Analysis of SREBP1 Target Genes

**Figure 3:**
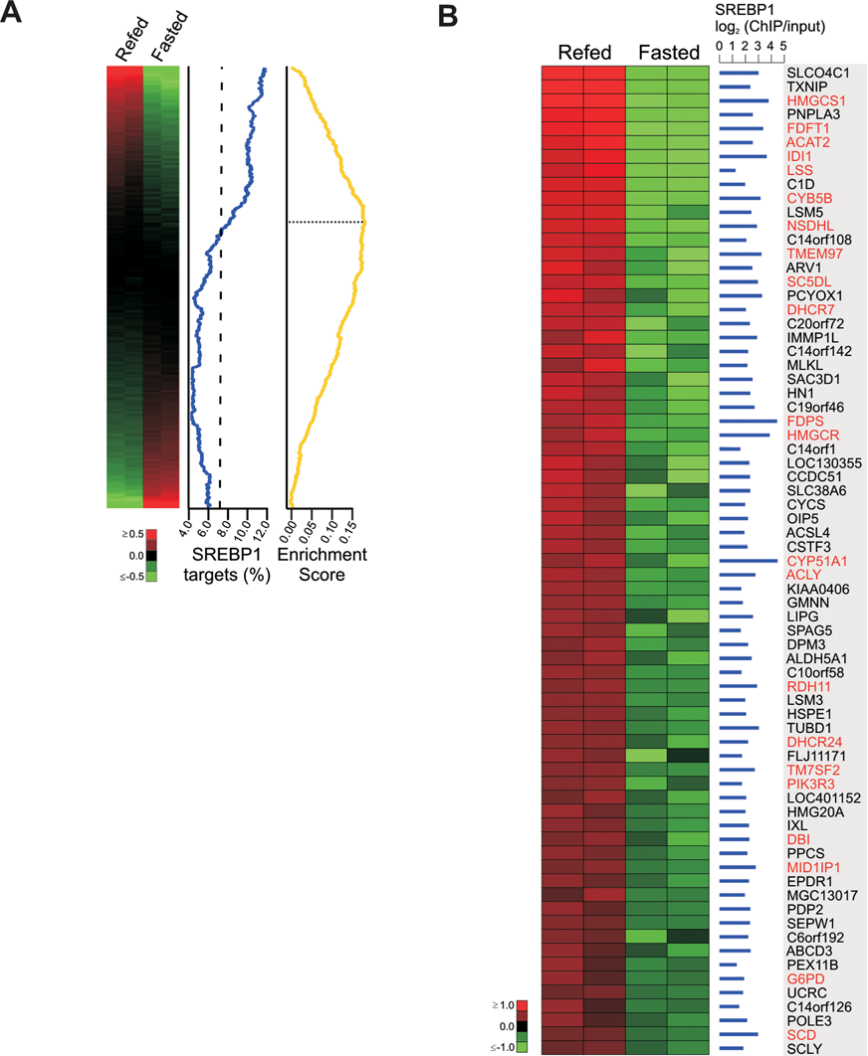
Gene expression changes of SREBP1 targets in fasted and refed HepG2 cells. A) Correlation of ranked gene expression data with SREBP1 occupancy. Left panel, replicate expression measures of 14,857 genes ranked by fold induction in the refed state are displayed as a heatmap. Middle panel, the corresponding percentage of genes occupied by SREBP1 in a 2000 gene sliding window. The dashed line indicates the expected percentage (i.e. the overall percentage of interrogated genes occupied by SREBP1). Right panel, GSEA [27] enrichment score plot. The enrichment score is a running sum statistic that, beginning with the top-ranking gene, increases when an SREBP1 target gene is encountered and decreases otherwise [27]. Enrichment of SREBP1 targets at the top of the ranked list results in a large positive deviation of the enrichment score from zero, indicated by the dotted line. In total, 555 out of 1066 represented SREBP1-occupied genes are located above this line and account for the significant (*P*<10^−3^) association of SREBP1 targets with the top of the ranked list. B) Detailed view of 71 SREBP1 target genes exhibiting greater than 1.75-fold higher expression in the refed state. Replicate expression measures are shown as a heatmap with the corresponding SREBP1 enrichment from ChIP-chip and gene symbol displayed on the right. Gene symbols of known SREBP1 target genes are indicated in red.

In the liver, the expression of many known SREBP1c target genes is downregulated in the fasted state when SREBP1c levels are reduced and upregulated upon high carbohydrate refeeding when SREBP1c levels increase [10]. To determine whether the putative direct targets of SREBP1 identified by ChIP-chip are differentially expressed in the fasted and refed states, we performed gene expression analysis. Levels of mRNA were compared between HepG2 cells treated with insulin and glucose (in the same manner as cells used for ChIP-chip analysis) or with forskolin and dexamethasone—molecules that reproduce the counterregulatory effects of glucagon and cortisol in the fasted state. The gene expression changes induced by these treatments were consistent with those observed in the fasted and refed liver in vivo (Table S7). To compare the list of SREBP1 targets with the gene expression dataset, the expression dataset was rank-ordered by fold change such that the most highly-induced genes in the refed state were at the top of the ranked list. We observed a marked enrichment of SREBP1 targets among the top ranking genes within this ranked list (Figure 3A). To determine whether this biased distribution of SREBP1 targets was statistically significant in comparison to the distribution expected at random, we used Gene Set Enrichment Analysis (GSEA) [27]. GSEA revealed that the enrichment of SREBP1 targets at the top of the ranked list was statistically significant (*P*<10^−3^) and identified a subset of 555 SREBP1-occupied genes that accounted for this effect (Figure 3A and Table S8). To highlight the most strongly induced SREBP1 targets in this subset, all SREBP1-occupied genes exhibiting greater than 1.75-fold induction in the refed state (71 total) are shown in Figure 3B; notably, 31% of these genes are known targets of SREBP1. These observations strongly indicate that transcriptional activation is the predominant effect of SREBP1 binding. Although SREBP1 binding sometimes results in gene repression, as previous studies have demonstrated for a few genes such as *IRS2* [23] and *PCK1* [28], repressed targets are less frequent than induced targets and fail to exhibit significant enrichment in our dataset. Importantly, our results demonstrate that a substantial proportion of the direct targets of SREBP1 identified by ChIP-chip are activated by insulin and glucose in the refed state.

### Combinatorial Regulation of Distinct Classes of Genes by SREBP1 and Its Partners NFY and SP1

**Figure 4:**
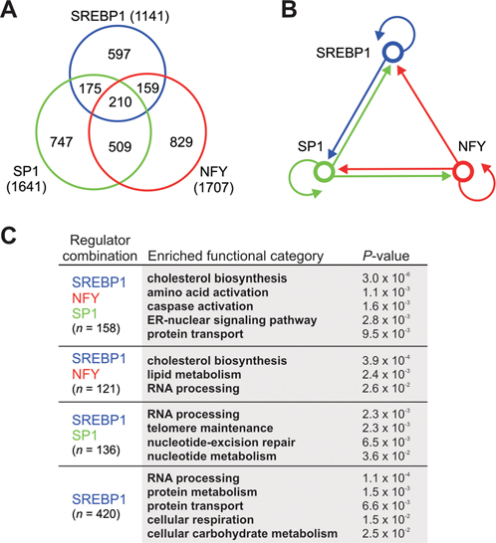
Genome-wide co-occupancy of SREBP1 with network partners NFY and SP1. A) Venn diagram showing the overlap among the sets of SREBP1-, NFY-, and SP1-occupied genes. B) Network diagram of the regulatory interactions observed among SREBP1, NFY, and SP1. Arrows indicate direct binding of one regulator to the promoter of another regulator or to its own promoter. Arrows to SREBP1 were determined by ChIP-qPCR with primers for the *SREBP1c* promoter as this promoter was not represented on the promoter array. C) Combinatorial regulation of SREBP1 target genes. Enriched GO categories were identified in each set of SREBP1 target genes occupied by a distinct combination of factors (or by SREBP1 alone) using BiNGO [18]. The number of genes with GO annotation within each set is indicated in parentheses. Representative enriched GO categories and their *P*-values are displayed for each regulator combination; the entire set of enriched GO categories for each combination is listed in Table S9.

To begin to investigate the regulatory networks in which SREBP1 operates, we performed location analysis with antibodies that recognize the transcription factors NFY and SP1—two key partners involved in the control of many SREBP1 target genes [7]–[9]. These ChIP-chip experiments were also performed in HepG2 cells treated with insulin and glucose as described above for SREBP1 and revealed that NFY and SP1 occupied the promoters of 1707 and 1641 genes, respectively (Tables S1 and S2). Comparison of these genes with the set of SREBP1-occupied targets revealed a high degree of overlap (Figure 4A); 32% of SREBP1 targets were occupied by NFY and 34% were occupied by SP1. In total, 48% of SREBP1 targets were occupied by at least one other transcription factor. The regulatory circuitry among SREBP1, NFY, and SP1 was highly interconnected (Figure 4B). For example, SP1 participates in multicomponent loops with both SREBP1 and NFY, an arrangement wherein two factors occupy each other's promoters. In addition, all three factors exhibited autoregulation, a hallmark of master regulators of key cellular processes [29]. Finally, these factors bind upstream of many other transcription factor genes, including the SREBP family member *SREBP2*, suggesting that SREBP1, NFY, and SP1 are involved in transcriptional regulatory cascades that can operate on additional target genes and pathways in an indirect manner.

We next used Gene Ontology to investigate the functions of genes targeted by different combinations of SREBP1 and its associated factors. This type of analysis is important for expanding our currently limited understanding of how transcriptional regulatory networks are organized into functional modules in mammalian genomes. Identification of enriched GO categories using BiNGO [18] revealed that different combinations of these regulators display distinct preferences for genes involved in certain biological pathways (Figure 4C and Table S9). In some cases, these pathways were targeted by a unique combination of regulators. For example, genes involved in lipid metabolism, including important lipogenic genes such as stearoyl-CoA desaturase 1, were preferentially occupied by the combination of SREBP1 and NFY (*P* = 2.4×10^−3^). Likewise, genes involved in carbohydrate metabolism (*P* = 1.5×10^−2^) and cellular respiration (*P* = 2.5×10^−2^), key nutrient-regulated pathways, were enriched among targets of SREBP1 alone, indicating that SREBP1 may cooperate with regulators other than NFY and SP1 at the promoters of these genes. In some cases, however, one biological pathway was targeted by multiple combinations of regulators. For example, genes encoding cholesterol biosynthetic enzymes were enriched among targets of all three regulators (*P* = 3.0×10^−6^) and among targets of both SREBP1 and NFY (*P* = 3.9×10^−4^). This type of configuration may reflect differences among genes in the same pathway, such as their timing or responsiveness to different stimuli. Additional representative enriched categories for each combination of regulators are displayed in Figure 4C. It will be of interest to determine how SREBP1 participates in the control of different biological pathways in combination with a wider array of transcriptional regulators in the liver by performing location analysis for additional factors, especially those that participate in nutrient-regulated pathways, such as SREBP2, LXRα, PPARα, and ChREBP.

In conclusion, these studies present the first global analysis of targets of an important set of regulators involved in mammalian lipid metabolism. The targets of SREBP1 are involved in a wide array of cellular processes suggesting new and expanded roles for this transcription factor. In addition, many of these targets are differentially regulated in the fasted and refed states, suggesting that they respond to changes in the diet. Finally, we find that SREBP1 and its associated factors NFY and SP1 form an interconnected regulatory circuit and bind in a combinatorial manner to functionally distinct sets of target genes.

## Materials and Methods

### Cell Culture

HepG2 cells were routinely cultured in DMEM supplemented with 10% FBS and 100 U/ml penicillin/streptomycin. For ChIP-chip and gene expression profiling, cells were cultured in glucose-free DMEM with 0.5% BSA for 12 h then treated with either insulin (100 nM) and glucose (10 mM) or with forskolin (1 μM), dexamethasone (1 μM), and pyruvate (2 mM) for 6 h in glucose-free DMEM with 0.5% BSA.

### Chromatin Immunoprecipitation

ChIP was performed as described with modifications [30]. Briefly, for each ChIP reaction, ∼5×10^7^ HepG2 cells were crosslinked for 15 min at room temperature, washed twice in cold PBS and swollen for 15 min on ice in hypotonic buffer. Nuclei were then pelleted and resuspended in lysis buffer for 30 min on ice followed by sonication using a Branson 450 Sonifier set at 50% amplitude. Samples were sonicated 8 times for 20 sec with 1–2 min on ice between pulses. Extracts were clarified by centrifugation at 14,000 rpm for 15 min at 4°C, pre-cleared with 50 μl of Protein A/G beads (Pierce) for 1 hr at 4°C, then incubated overnight with 10 μg of the appropriate specific or negative control antibody at 4°C. Antibodies used were rabbit anti-SREBP1 (sc-8984), rabbit anti-NFYA (sc-10779), rabbit anti-SP1 (sc-59), and normal rabbit IgG (sc-2027) obtained from Santa Cruz, and mouse anti-RNA Pol II (MMS-126R) obtained from Covance. Antibody-bound complexes were then captured by incubation with 50 μl of Protein A/G beads. Beads were washed once with RIPA buffer (150 mM NaCl), once with high-salt RIPA buffer (500 mM NaCl), twice with LiCl detergent, and once with TE. Antibody-bound complexes were then eluted by incubation with 100 μl of elution buffer (100 mM NaHCO_3_/1%SDS) for 15 min with gentle vortexing followed by a second 15 min elution with 150 μl of elution buffer. The eluates were combined and treated with RNaseA (20 μg/ml) for 1 hr at 37°C then incubated overnight at 65°C to reverse crosslinks. Whole cell extract DNA was also treated for crosslink reversal. Samples were then digested with Proteinase K (20 μg/ml) for 2 h at 37°C and DNA was purified by one extraction with phenol, one extraction with phenol/chloroform/isoamyl alcohol and one extraction with chloroform/isoamyl alcohol followed by ethanol precipitation and Qiagen PCR purification. Purified DNA was blunt-ended, ligated to universal linkers, and amplified using ligation-mediated PCR. Amplified DNA was then labeled with Cy3 or Cy5 dyes and hybridized to NimbleGen human promoter tiling arrays (array design “2005-04-18_HG17_min_promoter_set”) according to the manufacturer's protocol (NimbleGen Systems of Iceland).

### Promoter Tiling Array Analysis

Four biological replicate hybridization datasets (provided in Dataset S1) were generated for transcriptional regulators (ChIP), negative control rabbit IgG (control), and unenriched (input) DNA using an unpaired reference design. For SREBP1, NFY, and SP1, log_2_-transformed probe intensities were quantile normalized across the set of IP, control, and input replicate groups. For RNA Pol II, log_2_-transformed intensities were quantile normalized within groups then scaled to have a median feature intensity of 10. Normalized intensities were analyzed using a two-state Hidden Markov Model (HMM) in the program TileMap [11]. Briefly, TileMap first computes a test-statistic for every probe based on a hierarchical empirical Bayes model then uses HMM to combine information from neighboring probes and calculate the posterior probability that a probe is in the enriched state [11]. The enriched state was defined as: (ChIP>control) AND (ChIP>input). Enriched regions were selected by taking a posterior probability cutoff of 0.90 and merging probes separated by less than 500 bp. Regions less than 100 bp in length or containing fewer than 5 probes were discarded. A summary enrichment score was calculated for each region by taking the maximum probe value of mean(ChIP)-mean(input). Regions were matched to the closest RefSeq transcripts within 1500 bp on each strand based on the refGene table (hg17, May 2004 assembly) downloaded from the UCSC Table Browser (http://genome.ucsc.edu/) [31]. Entrez GeneIDs were assigned to transcripts via the refLink conversion table (hg17, May 2004 assembly).

### Target Validation

Quantitative PCR with site-specific primers was performed in duplicate on a BioRad MyiQ real-time PCR cycler with BioRad iQ SYBR Green supermix. Primers were designed for 19 randomly selected SREBP1 target promoters and for a negative control unbound region in exon 4 of the GAPDH gene. Normalized Ct (ΔCt) values for each sample were calculated by subtracting the Ct value obtained using input DNA from the Ct value obtained using SREBP1 ChIP DNA (ΔCt = Ct_ChIP_–Ct_input_). Fold enrichment was then calculated using the formula 2^−(ΔCt[target]−ΔCt[GAPDH])^. For the purpose of estimating the specificity of the SREBP1 ChIP-chip dataset, promoters with an average qPCR fold enrichment greater than 2 were considered enriched. Primer sequences are listed in Table S4.

### Motif Analysis

The motif discovery program MDScan [12] was used to identify candidate protein-DNA interaction motifs in the SREBP1 dataset. Input to the MDScan program consisted of 200bp sequences centered on the probe exhibiting maximum enrichment in each bound region. The top-scoring motif from MDScan was queried against the TRANSFAC database using the STAMP web server (http://www.benoslab.pitt.edu/stamp) [15] to identify the best matching known motifs. Results are presented in Figure S2. The DNA consensus sequence logos in Figure 2B were generated with the WebLogo tool (http://weblogo.berkeley.edu/).

### Functional Classification

Statistical analysis of the enrichment of Gene Ontology categories (‘Biological Process’ branch) was performed using BiNGO (http://www.psb.ugent.be/cbd/papers/BiNGO/) [18]. Enrichment was determined in reference to all Entrez GeneIDs annotated in the Biological Process branch (13,460 genes total). P-values are derived from a hypergeometric test followed by Benjamini and Hochberg false discovery rate correction. A P-value cutoff of 0.05 was used to identify significantly enriched categories.

### Gene Expression Analysis

Total RNA from 5×10^6^ fasted or refed HepG2 cells was collected using Trizol (Invitrogen) and further purified using a Qiagen RNeasy kit. Purified total RNA from two biological replicate experiments was then submitted to the Affymetrix Resource at the Yale W.M. Keck Foundation Biotechnology Resource Laboratory for labeling and hybridization to Affymetrix human genome U133 Plus 2.0 arrays. The robust multiarray averaging method [32] in the Bioconductor *affy* package [33] was used to generate expression measures from probe-level data. Updated probe set definitions were used to map expression data to the EntrezGene database [34]. For heatmap display, log_2_-transformed gene expression measures were ranked by expression difference (mean[refed]-mean[fasted]), mean centered, and visualized using Java Treeview (http://jtreeview.sourceforge.net/). The complete expression dataset is summarized in Table S7.

### Comparison of ChIP-chip and Gene Expression Data

To visualize the distribution of SREBP1 target genes within the ranked gene expression dataset, we generated a running overlap plot by sliding a 2000 gene window down the ranked list and calculating the percent overlap between the two datasets in each window. The significance of the association of SREBP1 target genes with the top of the ranked list was assessed using Gene Set Enrichment Analysis (GSEA) software [27]. Briefly, a maximum enrichment score (ES) was derived for the SREBP1 target gene set by taking the maximum deviation from zero of a running-sum statistic computed by walking down the ranked list and increasing the running-sum statistic when an SREBP1 target gene was encountered and decreasing it otherwise (as detailed in ref. [27]). The magnitude of the maximum ES reflects the degree to which a gene set is overrepresented at the extremes of the ranked list. The ranked list was then randomized 1000 times, calculating the maximum ES for each permutation in order to derive a null distribution. The nominal *P*-value for the SREBP1 target gene set (*P*<10^−3^) represents the fraction of scores in the null distribution that are at least as high as the observed maximum ES.

### Conservation Analysis

The set of SREBP1-bound DNA sequences (500 bp regions centered at the peak of ChIP enrichment) was scanned for matches to the PWM discovered by MDScan using the PatSearch web program (http://www.ba.itb.cnr.it/BIG/PatSearch/) with a similarity threshold of 0.80. The same procedure was performed for an equally-sized set of 500 bp regions selected at random from the promoters represented on the promoter array. The maximum 8-way phastCons conservation score [16] of each match in SREBP1-bound and random regions was then obtained using the Galaxy web server (http://main.g2.bx.psu.edu/). Scores from the SREBP1-bound and random regions were compared using a Wilcoxon rank-sum test.

## Supporting Information

Figure S1 Validation of SREBP1 binding by quantitative PCR. SREBP1 ChIP DNA was compared to input DNA to determine ChIP enrichment at 19 randomly selected SREBP1-occupied promoters. Bar heights represent qPCR enrichment at each promoter using site-specific primers. Error bars represent the standard deviation of replicate experiments. The dotted line indicates the fold enrichment cutoff used to determine whether a promoter was enriched or unenriched in the SREBP1 ChIP DNA. Primer pairs are listed in Table S3.
(0.02 MB PDF)

Figure S2 Motif similarity matches in the TRANSFAC database. The motif discovered by MDScan in SREBP1-bound sequences (Motif1) was compared to the TRANSFAC database using a motif comparison and alignment algorithm on the STAMP web server (http://www.benoslab.pitt.edu/stamp). The top 5 matches are shown, ranked in order of the significance (E value) of the pairwise alignment between the input motif and the TRANSFAC database motif.
(0.51 MB PDF)

Figure S3 Network visualization of enriched functional categories. Significantly enriched Gene Ontology categories (‘Biological Process’ branch) in the SREBP1 dataset are shown with their hierarchical relationships represented as a directed acyclic graph drawn using the BiNGO program (http://www.psb.ugent.be/cbd/papers/BiNGO/). Nodes are colored by P-value as indicated. Node size corresponds to the number of genes within each category. P-values are derived from a hypergeometric test followed by false discovery rate correction. A P-value cutoff of 0.05 was used to identify significantly enriched nodes. Some category labels are not shown for clarity.
(0.40 MB PDF)

Table S1 Chromosome coordinates of regions bound by SREBP1, POL2, NFY, and SP1. A summary enrichment score and position of peak enrichment are listed for each region. Results for each factor are displayed on separate worksheets.
(0.84 MB XLS)

Table S2 Binding sites of SREBP1, POL2, NFY, and SP1 matched to Refseq transcripts and their associated Entrez GeneIDs. The results for each factor are displayed on separate worksheets. Known SREBP1 target genes are highlighted in blue. A complete list of Entrez GeneIDs represented on the promoter array is also provided.
(2.96 MB XLS)

Table S3 Known SREBP target genes identified in a comprehensive literature search. The column titled “Liver target” indicates whether there is (1) or is not (0) evidence that a gene is targeted by SREBPs in the rodent or human liver. The column titled “SREBP1 ChIP-chip target” indicates whether or not a known target promoter was occupied by SREBP1 in our ChIP-chip dataset. Putative hepatic targets that are represented on the promoter array are shaded in gray. A literature reference for each regulatory interaction is also provided.
(0.06 MB XLS)

Table S4 Site-specific primers used for qPCR validation of SREBP1 binding sites.
(0.03 MB XLS)

Table S5 Results of BiNGO used to identify enriched Gene Ontology categories among genes occupied by SREBP1.
(0.08 MB XLS)

Table S6 SREBP1 target genes identified by ChIP-chip that are involved in cellular lipid metabolism based on Gene Ontology annotations.
(0.02 MB XLS)

Table S7 Results of gene expression analysis. Each row displays a gene represented by probesets on the Affymetrix expression array, the (log_2_) expression measures in refed and fasted HepG2 cells (two replicates each), and the overall expression difference (mean[refed]-mean[fasted]). Selected examples of genes exhibiting expression changes that are consistent with the physiological fasted and fed states are highlighted in blue.
(2.71 MB XLS)

Table S8 Results of Gene Set Enrichment Analysis. For each SREBP1 target gene, the corresponding rank and fold change in the Affymetrix fasted vs. refed gene expression dataset is indicated along with the value of the running-sum statistic (Running ES) at that point in the ranked list (see Materials and Methods). In addition, genes in the leading-edge subset (Core enrichment = Yes), which are responsible for the biased distribution of SREBP1 target genes in the ranked list, are highlighted in green.
(0.18 MB XLS)

Table S9 Results of BiNGO used to identify enriched Gene Ontology categories among genes occupied by different combinations of SREBP1 and its associated factors NFY and SP1. Results for each combination are represented on separate worksheets.
(0.08 MB XLS)

Dataset S1 Raw log_2_ probe intensities for each replicate hybridization of ChIP, control, and input DNA. The genomic location of each probe is indicated in columns 1–3.
(17.01 MB ZIP)
