# Supplementary figures and images for: Genome-Wide Occupancy of SREBP1 and Its Partners NFY and SP1 Reveals Novel Functional Roles and Combinatorial Regulation of Distinct Classes of Genes
Source: PLoS Genet. 2008 Jul 25;4(7):e1000133. doi: 10.1371/journal.pgen.1000133 (PMC2478640; doi:10.1371/journal.pgen.1000133)

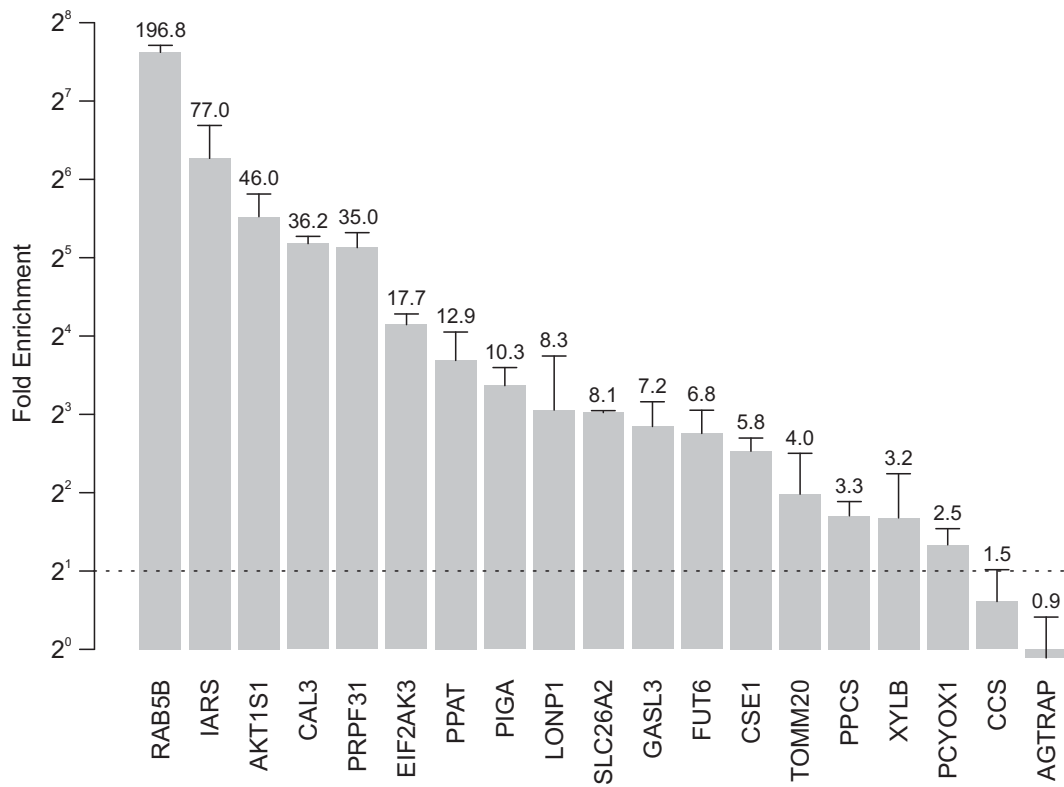

Supplement: Figure S1 — Validation of SREBP1 binding by quantitative PCR. SREBP1 ChIP DNA was compared to input DNA to determine ChIP enrichment at 19 randomly selected SREBP1-occupied promoters. Bar heights represent qPCR enrichment at each promoter using site-specific primers. Error bars represent the standard deviation of replicate experiments. The dotted line indicates the fold enrichment cutoff used to determine whether a promoter was enriched or unenriched in the SREBP1 ChIP DNA. Primer pairs are listed in Table S3. (0.02 MB PDF) [file pgen.1000133.s001.pdf]

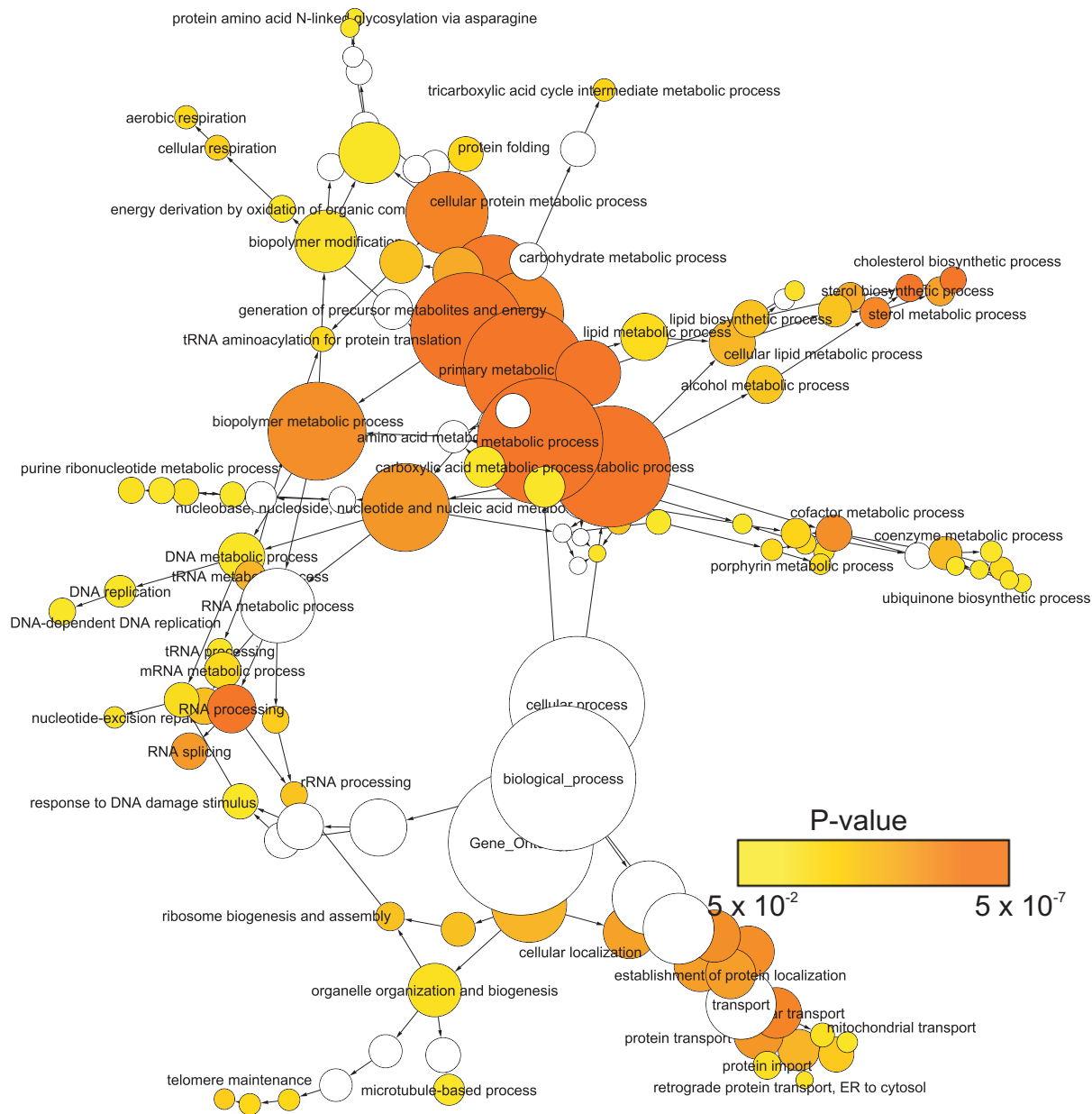

Supplement: Figure S3 — Network visualization of enriched functional categories. Significantly enriched Gene Ontology categories (‘Biological Process’ branch) in the SREBP1 dataset are shown with their hierarchical relationships represented as a directed acyclic graph drawn using the BiNGO program (http://www.psb.ugent.be/cbd/papers/BiNGO/). Nodes are colored by P-value as indicated. Node size corresponds to the number of genes within each category. P-values are derived from a hypergeometric test followed by false discovery rate correction. A P-value cutoff of 0.05 was used to identify significantly enriched nodes. Some category labels are not shown for clarity. (0.40 MB PDF) [file pgen.1000133.s003.pdf]
