# Supplementary material for: Genome-Wide Occupancy of SREBP1 and Its Partners NFY and SP1 Reveals Novel Functional Roles and Combinatorial Regulation of Distinct Classes of Genes
Source: PLoS Genet. 2008 Jul 25;4(7):e1000133. doi: 10.1371/journal.pgen.1000133 (PMC2478640; doi:10.1371/journal.pgen.1000133)
Supplement: Figure S2 — Motif similarity matches in the TRANSFAC database. The motif discovered by MDScan in SREBP1-bound sequences (Motif1) was compared to the TRANSFAC database using a motif comparison and alignment algorithm on the STAMP web server (http://www.benoslab.pitt.edu/stamp). The top 5 matches are shown, ranked in order of the significance (E value) of the pairwise alignment between the input motif and the TRANSFAC database motif. (0.51 MB PDF) [file pgen.1000133.s002.pdf]

Motif Similarity Matches

Motif1 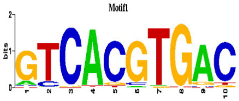

Name	*E value*	*Alignment*	*Motif*
SREBP-1_M00220	3.1680e-07	GTCACGTGAC<br>NTCACGTGAT	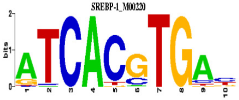
USF_M00796	9.3606e-06	GTCACGTGAC<br>STCACGTGNN	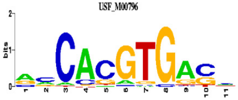
GBP_M00182	2.7010e-05	GTCACGTGAC<br>NNSACGTSNC	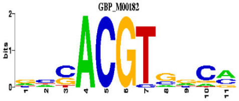
CBF1_M00303	5.0099e-05	TCACGTGAC<br>NCACGTGAY	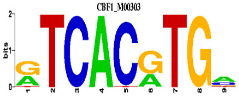
deltaEF1_M00073	5.8691e-05	GTCACGTGAC<br>NNCACCTNNN	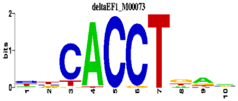
